# Understanding the Potential of Mental Health Apps to Address Mental Health Needs of the Deaf and Hard of Hearing Community: Mixed Methods Study

**DOI:** 10.2196/35641

**Published:** 2022-04-11

**Authors:** Judith Borghouts, Martha Neary, Kristina Palomares, Cinthia De Leon, Stephen M Schueller, Margaret Schneider, Nicole Stadnick, Dana B Mukamel, Dara H Sorkin, Dakota Brown, Shannon McCleerey-Hooper, Gloria Moriarty, Elizabeth V Eikey

**Affiliations:** 1 Department of Medicine University of California, Irvine Irvine, CA United States; 2 Department of Psychological Science University of California, Irvine Irvine, CA United States; 3 Department of Informatics University of California, Irvine Irvine, CA United States; 4 Department of Public Health University of California, Irvine Irvine, CA United States; 5 Department of Psychiatry University of California, San Diego La Jolla, CA United States; 6 Dissemination and Implementation Science Center Altman Clinical and Translational Research Institute University of California, San Diego La Jolla, CA United States; 7 Child and Adolescent Services Research Center San Diego, CA United States; 8 Riverside University Health System-Behavioral Health Riverside, CA United States; 9 Center on Deafness Inland Empire Riverside, CA United States; 10 Herbert Wertheim School of Public Health and Human Longevity Science University of California, San Diego La Jolla, CA United States; 11 The Design Lab University of California, San Diego La Jolla, CA United States

**Keywords:** mental health, deaf and hard of hearing community, mHealth, digital health, needs assessment, deaf, hard of hearing, hearing, focus group, survey, mixed methods, intervention, health app, user needs

## Abstract

**Background:**

Mental health concerns are a significant issue among the deaf and hard of hearing (D/HH) community, but community members can face several unique challenges to accessing appropriate resources.

**Objective:**

The aim of this study was to investigate the mental health needs of the D/HH community and how mental health apps may be able to support these needs.

**Methods:**

A total of 10 members of the D/HH community participated in a focus group and survey to provide their perspectives and experiences. Participants were members of the Center on Deafness Inland Empire team, which comprises people with lived experience as members of and advocates for the D/HH community.

**Results:**

Findings identified a spectrum of needs for mental health apps, including offering American Sign Language and English support, increased education of mental health to reduce stigma around mental health, direct communication with a Deaf worker, and apps that are accessible to a range of community members in terms of culture, resources required, and location.

**Conclusions:**

These findings can inform the development of digital mental health resources and outreach strategies that are appropriate for the D/HH community.

## Introduction

Accessing mental health services is a challenge in the United States, a challenge that is further magnified for persons who are deaf and hard of hearing (D/HH). D/HH is an umbrella term used to encompass a diverse community. Other terms used by members of the community may include “deaf,” “Deaf,” or “late-deafened.” Following feedback on terminology from our participants, we have chosen to use the term D/HH throughout this paper to refer to this community, and we acknowledge that participants may use different terms to self-identify. Debate exists within the community over Deafness or deafness as disability versus Deafness or deafness as linguistic minority. While this is beyond the scope of this paper, we encourage readers to see Skelton and Valentine for an overview [1]. The D/HH community, which has often been referred to as an “invisible minority” [2], is a community with its own unique culture, traditions, and challenges. Members of the D/HH community may face significant psychosocial challenges and environmental adversity as they navigate an inherently ableist, hearing world [1,3]. Several studies do show that members of the D/HH community experience higher rates of psychological distress [4-6].

When it comes to accessing care, the D/HH community faces significant health care marginalization and health care inequities [3]. D/HH individuals report a lack of availability of mental health services [7], and Critchfield [8] estimates that 80% to 90% of people who are D/HH with severe and persistent mental illness do not receive care. As summarized by Pertz et al [3], this lack of mental health care access is multifaceted and largely stems from systemic barriers facing the community, for example, insurance coverage [9], lack of interpreters for health care visits [10,11], and lack of evidence-based, culturally competent mental health treatment options [12]. Patient outcomes for D/HH persons are better when they receive care from providers who understand Deaf culture, but these are rarely available [13,14]. Pertz et al [3] found that Deaf signers at an integrated medical and behavioral health program with a telemental health (TMH) intervention reported significantly lower depression and anxiety scores from baseline and high satisfaction ratings due to accessible communication and optional ongoing care through a TMH platform. Negative experiences and challenges communicating with ineffective providers can impact treatment engagement and adherence [15], creating general distrust, reluctance, or resistance to the mental health care system [16].

Technology provides opportunities to overcome some of the barriers to accessing mental health care traditionally facing the D/HH community. Many people in the D/HH community report using technology in other aspects of their lives. Examples include text-to-speech apps or smartphone features, such as Ava or Siri; videoconferencing, which is commonly referred to as videophoning in the D/HH community; sound enhancement apps, such as Sound Amplifier; and a variety of visual alert assistive technologies [17]. A national survey by Maiorana-Basas and Pagliaro [18] suggests that technologies such as texting, emailing, and instant messaging are used at similar rates across the population, regardless of hearing status. The rapid development of technology has led to a proliferation of digital resources designed to support and help people manage their health, and TMH services are effective treatment modalities among the general population (see Langarizadeh et al [19] for a review). TMH services are especially suited in the treatment of D/HH persons because the D/HH community may already have a level of familiarity with visually oriented technologies and assistive technologies, which may help facilitate treatment delivery [20]. TMH may also help facilitate service delivery to D/HH individuals who may otherwise not have a local, culturally competent mental health provider from whom to seek treatment [16]. Furthermore, smartphone access in the United States is increasing, although it is not ubiquitous, and several socioeconomic factors influence access, particularly considering that the average price of a smartphone is now over US $500 [21]. Those with technology access and digital literacy skills are likely to be younger, highly educated, and possess adequate financial resources. Although technology is often posited as the “great equalizer,” it can also serve to further widen the gaps between privileged and underprivileged groups, who differ in their access to, knowledge of, and ability to make full use of the medium [22].

Mobile health (mHealth) apps for the D/HH community exist, though these largely serve as assistive technologies that aim to augment people’s ability to navigate and communicate in public and with family and connect with other members of the community. In Romero et al’s [23] review of existing mHealth apps for the D/HH community, only two apps from an initial search list of 217 apps were related to mental health. They note that the relatively low yield and high turnover of available apps necessitates more development of apps for the D/HH population. There are no studies, of which we are aware, that have explored mental health apps specifically. Indeed, in our own searches of available resources to inform the development of this study’s methodology, we did not identify any apps to support the mental health of the D/HH community.

In general, while previous studies have identified several challenges among the D/HH community to access mental health resources, it is less understood if and how mental health apps may overcome these challenges. The aims of this study were to explore the mental health needs of the D/HH community and explore how digital resources such as apps may be able to support these needs. To address these aims, we conducted a focus group with 10 community members to get an in-depth understanding of their experiences and perspectives.

## Methods

### Overview

A community-based participatory approach was used throughout our study to engage community members in multiple stages of the study. The effort ensured that the data collection content and processes were appropriate, the study design was suitable, and the voices of community members were accurately represented in reporting our findings.

### Participants

A total of 10 people participated in one focus group, and 9 of these participants also completed a follow-up survey. Participants were members of the Center on Deafness Inland Empire (CODIE) team and based in Riverside County, California. The CODIE team comprises people with lived experience as members of the D/HH community. CODIE works to advocate for the community by empowering individuals with information, offering training and opportunities, and working to resolve challenges in areas such as communication barriers, peer counseling, independent living skills, community education, and outreach. Participants were invited by email to participate in the focus group by a lead advocate on the CODIE team.

Demographic information was collected using a web-based English-written survey distributed after the focus group. All participants reported comfort with written English, and the survey was developed in partnership with the CODIE team and Riverside County. One participant did not complete the survey, so demographic details describe 9 participants. Given the small sample size, we report the general characteristics of the sample. Participants ranged in age from 30 to 60 years (mean 44.1, SD 11.3). Participants reported their gender as female and identified as White, Black or African American, Asian, American Indian or Alaska Native, or Mexican, or they identified with more than one race. Out of 9 participants, 8 (89%) most often used American Sign Language (ASL) at home, and 7 (78%) participants reported their preferred communication method as ASL.

### Measures

For the focus group, the research team developed a focus group facilitator guide with discussion topics and sample questions. Topics and questions were developed and refined based on research partners’ interests and their past learnings working with the D/HH community. The research partners consisted of staff from Riverside County Behavioral Health, peer specialists, and the lead advocate on the CODIE team. The research team met with evaluation staff from Riverside County Behavioral Health prior to the focus group to review the questions included in the guide, obtain input on the topics covered, and ensure language used was appropriate and understandable. The lead advocate also provided best practices for facilitating focus group discussions with the D/HH community. First, it was important for the facilitator to have a clear video picture in a well-lit room, tie long hair back, and minimize distractions such as moving objects in the background, so that participants could focus on body language, facial expressions, and lip movements. Second, it was advised for the facilitator to look directly into the camera and speak slowly and clearly to allow for lip reading and interpretation. Third, the facilitator should pause after asking a question to allow for interpretation and look at the interpreter to ensure that interpretation had occurred. Lastly, interpreters should introduce themselves at the beginning of the focus group and provide guidance for participants to pin them on their screen. While these practices were given specifically for a virtual focus group, many of them are applicable for in-person focus groups too, such as speaking slowly and creating pauses for interpretation (see Balch and Mertens [24] for further lessons learned from D/HH participants on conducting focus groups).

The main focus of this study was on understanding the mental health needs of the D/HH community and how mental health apps may support the D/HH community’s mental health needs. Therefore, topics included perspectives on both mental health in general and mental health technologies specifically. Topics covered in the focus group guide included the following:

- Perspectives on mental health within the D/HH community
- Mental health needs and services available for the D/HH community
- Use of and attitudes toward apps and technologies for mental health within the D/HH community
- Challenges and facilitators to using mental health apps and technologies by the D/HH community.

The follow-up written survey was sent 5 days after the focus group. The survey asked additional questions around digital mental health and was intended to supplement findings from the focus group as well as allow participants to express thoughts outside of the focus group setting. The survey questions were developed before the focus group but were refined based on information obtained in the focus group. For example, one survey question asked what aspects of mental health apps were important to participants; the answer options of this question were updated to include certain aspects mentioned during the focus group. The four topics covered in the survey are discussed next.

The first topic was “barriers to mental health resources.” Participants were asked to report all barriers, if any, they faced to accessing mental health–related resources. They were instructed to “select all that apply” from a list of options, type free text, or both. The list of barrier options was adapted from the Healthy Minds Study, an annual web-based survey assessing mental health and service use among college students [25].

The second topic was “important aspects about mental health apps.” Participants were asked to rate the extent to which different aspects of mental health apps were important to them (eg, “The app is free”). They were asked to rate items on a scale from “not at all important” (1) to “extremely important” (5).

The third topic was “mental health app use.” A single question was used to identify whether participants had used mental health apps. In the survey, a mental health app was defined as “an application on your mobile phone or tablet device that helps you manage your mental, emotional, or psychological health or get access to resources to support your mental, emotional, or psychological health.” Participants could select whether they had used apps in the past, were currently using apps, had never used apps but would be interested, or had never used apps and were not interested. Participants were also asked to rate three statements related to whether they had the resources required to use mental health apps (eg, “I have the resources necessary to use mental health apps”). The scale ranged from “strongly disagree” (1) to “strongly agree” (5). The items were based on the facilitating conditions subscale of the unified theory of acceptance and use of technology questionnaire [26]; they were adapted to refer to mental health apps specifically.

The fourth topic was “current and desired resources to support mental health.” Participants were asked to select what resources they currently used and what strategies they wished to use to support their mental health, if any (eg, “informal support, such as talking with or spending time with family or friends”). They were instructed to “select all that apply” from a list of options, type free text, or both.

The complete survey instrument is included in Multimedia Appendix 1.

### Procedure

The focus group took place on September 11, 2020, and survey data collection took place between September 16 and 28, 2020. The focus group was held online via Zoom (Zoom Video Communications, Inc), facilitated by hearing research staff, and supported by two interpreters to translate spoken English into ASL and vice versa. Each focus group question was also shown in written English in the chat window of the Zoom session, and participants were able to provide written responses in the chat window. The focus group discussion was audio recorded. The audio recording captured both the ASL translated to spoken English and the chat messages, which were read aloud. The duration of the focus group was 2 hours. The survey was distributed via Qualtrics and took approximately 20 minutes to complete. Participants received a US $30 gift card for their participation in the focus group and a US $10 gift card for completing the survey.

### Ethical Considerations

The study was approved by the University of California, Irvine, Institutional Review Board (IRB; review number No. 20195406). Prior to the focus group, participants were emailed a study information sheet that was reviewed and approved by the CODIE lead advocate and the IRB. The sheet was then reviewed in the focus group session, with an opportunity for participants to ask questions. Participants were asked for their permission to audio record the conversation at the start of the focus group.

### Analysis

The audio recording of the focus group was transcribed. The analytical framework used to analyze the transcript was the six-phased approach of thematic analysis as described in Braun and Clarke [27], which involves the following: (1) familiarizing yourself with the data, (2) generating initial codes, (3) searching for themes, (4) reviewing themes, (5) defining and naming themes, and (6) writing up the analysis. We adopted an interpretivist epistemological position and used an inductive analysis approach: there was no pre-existing coding scheme, and codes were created based on what emerged from the data. The qualitative analysis software Atlas.ti (version 22.0.1; Scientific Software Development GmbH) was used to code the transcript. The initial coding (phases 1-3) was done by one of the non-D/HH researchers, who is a trained PhD researcher with expertise in user experience and thematic analysis. For phase 4, a preliminary summary of findings was shared with study participants and other members of the D/HH community and research team who attended the focus group in order to check that this was what was said, to corroborate, to correct or extend interpretations of findings, and to further refine themes. These findings were discussed over email and during a video call meeting, where an interpreter was present to support the discussion. For phase 5, themes were defined and named by the non-D/HH research team. For phase 6, a draft of the write-up was shared with members of the D/HH community and research team who attended the focus group to provide feedback, craft the language, and add details.

We analyzed the survey data using descriptive statistics in the form of the number of people who selected certain answers. The statistical software SAS (version 9.4; SAS Institute Inc) [28] was used for analysis of the survey data. The main purpose of the survey was to supplement findings from the focus group and describe the study sample (eg, demographic information, the number of participants who had used mental health apps before, and the number of participants who wanted to use certain mental health app features that were discussed during the focus group).

## Results

The following section presents an overview of study results. Unless otherwise specified, results are based on the focus group. Illustrative quotes are provided for each theme.

### Current and Desired Strategies to Support Mental Health

Participants had not used any mental health apps before. A total of 6 out of 9 (67%) participants indicated on the survey that they were interested in using one, and 3 (33%) participants indicated they were not interested. Though mental health apps were not commonly used, participants shared that they used other online resources to support mental health, such as spiritual classes, meditation, and ASL yoga:

I use deaf spirituality. That kind of covers a lot of different things. Healing, holistic healing. There’s meditation. I use a website as well as a good resource, oh, to find practitioners who use sign language for all of those types of things.

Other apps that were reported to be used were communication apps to connect with others, such as WhatsApp, Zoom, and Skype.

Participants were asked on the survey what their current and desired strategies were to support their mental health. The most common strategies currently used involved informal support connecting with friends or family (n=6, 67%), peer support (n=6, 67%), and use of social media (n=5, 56%). The most common desired resource was professional mental health services (n=6, 67%), followed by activities like writing, painting, and playing or making music (n=5, 56%); online forums or communities (n=4, 44%); and websites (n=4, 44%). A total of 3 (33%) participants reported wanting to use online chat and peer support, as well as exercise programs or activities to manage their mental health.

Participants were also asked on the survey about what the broad D/HH community would like to be able to do with mental health resources. They felt the D/HH community would be most interested in resources that allow them to talk with other people to give and get support (n=6, 67%) as well as those that allow them to express themselves or have an outlet through art, photos, or writing (n=6, 67%). A total of 5 (56%) participants also reported several other possible interests, such as identifying or recognizing symptoms, working through negative emotions and thoughts, connecting with a professional, and getting information about how to cope with stress, grief and loss, trauma, and relationship issues.

### Challenges to Using Mental Health Apps

#### Support for a Spectrum of Language and Linguistic Needs Within the Community

The most reported barrier to accessing mental health services on the survey and during the focus group was the difficulty in finding mental health care providers that knew ASL. Similarly, the main barrier to using online mental health tools specifically, as reported on the survey, was difficulty finding a tool that supported ASL.

Beyond a lack of ASL support, participants reported issues to accessing mental health services with respect to communication, access, and feeling welcome. Participants shared that there are a range of language and linguistic needs within the community, with some people feeling more comfortable with English, whereas others are more comfortable with ASL. Furthermore, there are different literacy levels within the community in terms of understanding English. Participants recommended providing different options to present content through a digital mental health intervention, such as text, videos, and icons, and providing ASL video where possible. One participant noted the following:

Talking about English and ASL, there’s neither one that is better than the other. It’s a matter of what the person feels most comfortable with...You also don’t want it to be just ASL only, it might force somebody out of their comfort zone. So we need to consider that spectrum of language and linguistic needs and comfort levels, which is really wide.

In the context of differing linguistic needs, participants also discussed knowledge gaps in relation to mental health concerns. The D/HH community misses incidental learning opportunities around mental health, which happen when people gain knowledge from informal interactions and overhearing conversations that can be related to societal changes in attitude toward mental health. These learning opportunities typically rely on spoken language.

#### Lack of Accessible Services

Even if an interpreter could be provided to aid communication with a mental health provider, participants reported that many community members would not feel comfortable with having an interpreter present and would feel safer if they could speak directly to a mental health provider with the same language. One participant explained the following:

Sometimes interpreters will use different word choices and it’s not what I mean. Or you know, confidentiality, because people may want to keep that privacy.

One participant mentioned that to build a connection with a health provider, it helps to talk to someone who looks and signs like them. An additional barrier to accessing mental health services was that providers were not sensitive enough to cultural differences. For example, a participant explained that hearing providers do not have a “deaf heart” and the sensitivity or the same experiences as them.

Participants said there was a lack of Deaf workers in the mental health profession and that it was challenging to find mental health services for the D/HH community. Participants expressed concerns that there was a lack of accessible resources overall and for specific services, such as marriage counseling, anger management, substance abuse treatment, and support for domestic violence. For services that were available, participants said that community members were sometimes limited in terms of their insurance and what services they could access. For example, one participant commented that services may only be available out of their state and, thus, not covered by insurance, or that the only ASL services available are very basic.

#### Stigma Around Mental Health

There was a consensus during the focus group that stigma around mental health was a considerable challenge in the community, and that community members did not want other people to know they were accessing mental health services. Participants had concerns over the use of the term “mental health” and said that positive and uplifting terms centered around spirituality and healing would be more appropriate, stating that these would resonate more with the community and signal that a positive experience is forthcoming. For example, one participant suggested the name “Healing Hands” for a mental health app.

Participants expressed concerns that for many community members, miscommunication has had negative repercussions in the past, which can increase stigma, and there are fears of experiencing negative side effects of getting treatment. For example, members with children may have fears that if they talk about their mental health challenges, their family, such as their children, may be taken away or there may be financial consequences. One participant stated that mental health can be perceived as “just another thing wrong with my head.”

Participants noted that community members may have privacy concerns around the use of mental health services, such as concerns around whether their information was going to be safe. On the survey, 3 (33%) participants indicated that they had privacy concerns on their personal information being visible by using mental health apps.

Participants expressed a need for increased education and awareness around mental health, and to promote a message that mental health services are helpful in a good way, that it was okay to seek help, and that mental health is for everyone:

Nowadays what I’m seeing is Deaf and Hard of Hearing people putting vlogs emphasizing it’s okay to feel whatever you’re feeling, and it’s okay to look for help. And I think that’s key, if developing an app...to emphasize that. That’s what most of society is doing at this moment.

### Facilitators to Engagement With Mental Health Apps

#### Overview

Participants gave several recommendations on marketing a mental health app to the D/HH community. Examples included using posters and signs, scrolling and video advertisements for the app at medical offices and social service offices, contacting nonprofit organizations that service the D/HH community, and word of mouth. It was important that the marketing materials supported a feeling of being welcome, for example, through visual advertisements that showed the step-by-step process of using the app. Participants preferred the app to be advertised with ASL people signing, using more visuals than words. Participants pointed out that instead of an “interpreter” sign, a better sign would be the two-handed sign for “peer,” “advocate,” or “support,” ideally with hands of different colors and genders. They also approved of the “same same” sign.

With respect to speaking with a health provider, participants expressed a need to be able to choose a specific person with whom they felt comfortable talking. Some people may have experienced trauma with past providers and wanted to talk to someone who would be a good fit for them regarding language and other characteristics, like gender. It was important to have diversity within the community and presented on an app, in order to make the app accessible to everyone.

Lastly, if a mental health app were to be developed that was inclusive of the D/HH community, participants expressed a preference for an app that would be useful to everyone, not just members of the D/HH community. Clicking on an app that would be specifically labeled for the D/HH community could give a feeling of being singled out. As one participant explained:

We want to try and keep that general to have access to things instead of feeling like, ‘oh okay, I have to click on this because it says deaf,’ that singles me out.

#### Immediate and Continuous Access to Resources

Participants placed importance on the fact that a mental health app should be accessible to a range of people in terms of language, culture, resources required to use the app, and location. They explained that community members may have limited data or memory on their phone, no access to high-speed internet, or no access to a computer. A mobile app was the preferred platform to enable people to access resources on the go. On the survey, 5 (56%) participants indicated that they had concerns about their mobile data plan when using their mobile device, 4 (44%) participants did not have the necessary resources to use mental health apps, and 3 (33%) participants were concerned about having enough space to download apps on their smartphone.

Participants also expressed value for an app to provide immediate access to resources and services. At the time of the focus group, people had to go through a long intake process before they could connect to mental health services:

Having that immediate assistance, to somebody live or whatever it is, right there is important rather than having to go through all of these different things...you have to go through all of that demographic information and you have to basically tell your life story before you can get to somebody.

Given the range of literacy levels within the D/HH community, participants worried that people may not understand all intake questions, which can slow down the process further and reduce interest in engagement. A participant mentioned that having the intake available in both English and ASL would likely make the intake process go smoother.

Participants also said that it would be ideal to have unlimited access to resources, as opposed to there being a limit to the number of times they could access them. Especially during the global COVID-19 pandemic, services were sometimes used by people just to connect and talk to someone, and some consumers accessed mental health services multiple times a day.

Though participants were part of a local community-based agency, they named several benefits of making an app globally accessible to anyone, rather than tying it to a specific location. First, people may be located elsewhere but prefer coming to a specific organization, such as CODIE, for services. Second, there were benefits to working together with other organizations. If someone is in need outside of standard business hours, there may not be anyone near them to help, but there may be someone awake in another time zone and location who can provide support. Third, these collaborations could facilitate linking to resources from other organizations, in order to spread awareness and help the broader D/HH community. Participants suggested making apps available to people from Gallaudet University, the only university in the world where students learn in ASL and English.

#### Important Features for Mental Health Apps

Participants were asked on the survey what the most important aspects were that mental health apps should offer. The most important aspects were suicide prevention support, emergency support, peer support and chat, and telehealth (ie, referring to a direct connection to clinical mental health services within the app). A chatbot was rated as the least important.

During the focus group, participants reported that it was important for people to interact with a human and not an avatar*.* An avatar is a computerized figure, such as an icon, that can represent a simplified figure of a person. The term avatar can refer to different things and can also be used for digitally created characters that turn speech into sign language. Participants’ reservations about an avatar were that it lacked facial expressions and body language, which are important to have in addition to signs in communication. One participant explained as follows:

We want an actual person, not an avatar because an avatar lacks that body language, those expressions that, a lot of people that maybe are at the gestural language level would understand the body language more than the actual signs.

Furthermore, they reported that it was valuable to build a real relationship with a human being. It was not discussed during the focus group whether participants did not want avatars at all or whether avatars could be used to supplement interaction in an app, if it also allowed connection with a live person.

Participants mentioned past use of Deaf clubs, which are places where Deaf people can meet face-to-face and socialize [29]. Participants shared that they used Deaf clubs for socializing, fun, and games, and expressed the desire to see these Deaf clubs being used for mental health support. It was discussed whether an app could have something similar to a Deaf club.

#### Inclusion of Diverse Community Members in Technology Development

To reduce stigma around seeking help for mental health, participants suggested having members of the community contribute to an app, for example, through blogs or short videos to share their experiences and knowledge. Participants liked the aspect of inclusivity where visitors can be “part of the news”:

Like a blog that people could add—share their experiences and their knowledge and their education...They could be a part of the news basically. Instead of watching the news with captions, they could watch this person signing that.

In addition to providing content, participants recommended having community members involved in the design process to give feedback on features and on what can be improved. Some participants reported that it would also be valuable to have community members provide guidance on how to access and use the app, for example, through instruction videos and visual posters with step-by-step instructions.

## Discussion

### Principal Findings

The aim of this paper was to understand the mental health needs of the D/HH community and how mental health apps may be able to support these needs. In line with previous work [7,9], participants indicated that community members had limited access to mental health resources. Digital solutions, such as mental health apps, may increase access to resources, but our study highlighted that it is important to take certain factors into consideration to facilitate engagement with such apps.

Some of the themes we found are common barriers among other mental health–seeking populations as well, such as stigma [30]. This barrier may be exacerbated in the D/HH community due to missed incidental learning opportunities about mental health. Furthermore, similar to previous work with hearing populations [31], participants valued immediate access to resources. Participants rated access to suicide prevention support and peer chat as one of the most important features to include in a mental health app, which resonates with work with other communities: a recent study with essential workers found that one of the most desired features for a mental health technology was the ability to chat with a mental health professional or peer, and a link to mental health resources and crisis support [31].

Factors that may be more unique to the D/HH community are the need for both ASL and English support, and the finding that participants wanted a general app that is inclusive of the D/HH community, rather than an app exclusively made for them. For example, participants emphasized the importance of including members of the D/HH community on the app, but to market an app as usable for everyone to avoid singling out the D/HH community. This finding further supports the need for customization and personalization of mental health apps [32,33] and the importance of inclusive design and designing for a wider population [34]. The ability to customize an app to a user’s personal needs can facilitate feelings of perceived fit to a user’s culture and values [35], without singling out a particular community.

While further follow-up studies are recommended to corroborate themes with a larger population, initial takeaways can be extracted from our findings to inform creation and development of digital mental health resources, such as apps. Below we outline several learnings that may be important to consider when developing digital mental health resources for the D/HH population.

### Support for a Spectrum of Language and Linguistic Needs Within the Community

Similar to previous work [10,36], the greatest barrier to accessing mental health services identified by participants pertained to communication issues. Participants reported a lack of Deaf workers and mental health care providers that knew ASL. It is, thus, important to support a spectrum of linguistic needs within the community.

Participants in our focus group primarily highlighted the limitations of English-language mental health services, rather than positive experiences. A previous focus group with D/HH community members indicated that there may be social pressure during ASL focus groups that limits participants from sharing any positive experiences with English health care communication [37]. While our study participants acknowledged that some community members may prefer English, the main issue was that there needs to be support for a *range* of linguistic needs, rather than English or ASL support alone. Participants shared that there are various language and linguistic needs within the community, with some people feeling more comfortable with English and ASL, and there are different literacy levels in terms of understanding English. Our survey further found that a lack of Deaf workers in the mental health profession was not the only barrier to accessing resources, but it was the most common.

While previous studies found difficulties in accessing mental health resources due to a lack of interpreters [37,38], participants in our study indicated that the availability of an interpreter alone may not be sufficient. Even with an interpreter present, participants explained that community members may not feel comfortable talking via an interpreter and prefer to communicate with a Deaf worker directly. This sentiment is consistent with findings from Steinberg et al [39] who found a preference for health care practitioners who are fluent in ASL and support from other Deaf individuals.

To support a range of linguistic needs, participants recommended providing different options for presenting content through an app, such as text, videos, and icons, and providing ASL video where possible. Participants also provided marketing suggestions to support a feeling of being welcome, for example, through visual advertisements that show the step-by-step process of using the app.

### Stigma and Appropriate Use of Terminology

Similar to prior studies with D/HH community members [39], study participants expressed that there was stigma within the community around mental health issues and seeking help for these issues. Though stigma can be a common barrier among the population in general [30], it may especially be an issue for the D/HH population, as they are not exposed to mental health issues and information in the same way as the general public [38]. Even though study participants expressed a need for more Deaf workers in the mental health profession, previous work found that because of this lack of exposure, Deaf workers may be less knowledgeable about mental health issues [38].

Prior work on mental health apps has suggested that delivering support through technology can overcome stigma barriers, as people do not need to know one is seeking help [40]. However, participants in our study still had concerns about their information not being private through an app. To help mitigate privacy concerns, it is important to be transparent on how app data are collected and stored and how they will be used. Furthermore, participants recommended that instead of using the term mental health, positive and uplifting terms around healing are preferred in order to facilitate adoption of a mental health app.

### Education Around Mental Health

Communication issues can complicate accurate reporting of mental health prevalence in the D/HH community [41,42], and our findings further showed that D/HH members can often miss out on informal conversations and may not be as knowledgeable about mental health as the hearing population. This finding highlights that increased education around mental health may be especially important for this community. Participants expressed a need for increased education and awareness around mental health, for example, through short videos and by having members of the community share their experiences. Participants stated that there was a need for people to understand that mental health services can be helpful, and that strong mental health is a goal for everyone.

### Include Members of the D/HH Community and Market for Broader Community

Ideally, participants preferred to have direct communication with a Deaf worker that had the sensitivity and experience to communicate with members of the community. Participants also recommended involving community members in providing content and sharing feedback about improving app features. It was important to have an app that is inclusive of, but not exclusively for, the D/HH community. Participants preferred an app that would be useful for anyone and that would not just be focused on their community, which may exacerbate feelings of being singled out.

### Limitations and Future Work

The study has several limitations. First, care should be taken to generalize its findings to the broader community. An advantage of a focus group setting is that it has been shown to be a suitable methodology for Deaf culture to gather and share information in a safe setting [37,38], but sample size is limited. In addition, participants in the focus group were engaged with an advocacy group and involved in the community, so their experiences may be different than those of the general community. Participants had experienced hearing loss since birth or early in life, and their experiences may differ from those who experience hearing loss because of old age or those who experience hearing loss later in life. Our study offers insights into how mental health needs of the D/HH community may be supported through digital therapeutics, which would be worthwhile to explore further in a larger-scale study. Second, an English written survey was used to collect participants’ demographic information. Though all participants were able to read and write in English, the majority of participants’ preferred language was ASL, and one participant did not complete the survey. Our study results have since been used to inform a collaborative effort to create an ASL survey for broader needs assessment with the D/HH community. Third, results were collected during the COVID-19 pandemic, which may have increased mental health concerns and interest in mental health resources. Lastly, we used the English translation of the focus group discussion for data analysis. There may be limitations in using an English translation, as information may be filtered and expressions can differ from ASL. For example, personal pronouns in ASL are not gender specific [37]. To ensure that our analysis and findings accurately represented participants’ views, we refined our findings through member checking with the focus group participants who are members of the D/HH community.

### Conclusions

This study looked at the mental health needs of the D/HH community and how mental health apps may be able to support these needs. There was a need for more Deaf workers and ASL support to support a spectrum of linguistic needs; a need for increased education to reduce stigma around mental health; a need for an app that is accessible to a range of people in terms of culture, resources required, and location; and a need for immediate and unlimited access to resources. These findings are important to consider for the development and dissemination of mental health apps to meet the needs of the D/HH community.

## Appendix

Multimedia Appendix 1 Survey items.

## Abbreviations

ASL: American Sign Language
CalMHSA: California Mental Health Service Authority
CODIE: Center on Deafness Inland Empire
D/HH: deaf and hard of hearing
IRB: Institutional Review Board
mHealth: mobile health
TMH: telemental health

## References

[1] Skelton T, Valentine G (2003). 'It feels like being Deaf is normal': An exploration into the complexities of defining D/deafness and young D/deaf people's identities. Can Geogr.

[2] Hunt G, Pupo N, Glenday D, Duffy A (2010). Trapped in the shadows: The working lives of invisible minorities. The Shifting Landscape of Work.

[3] Pertz L, Plegue M, Diehl K, Zazove P, McKee M (2018). Addressing mental health needs for deaf patients through an integrated health care model. J Deaf Stud Deaf Educ Internet.

[4] Bridgman G, MacPherson B, Rako M, Campbell J, Manning V, Norman-Kelly T (2000). A national epidemiological survey of mental illness in the New Zealand Deaf community. Proceedings of the 5th European and 2nd World Conference on Mental Health and Deafness.

[5] Fellinger J, Holzinger D, Dobner U, Gerich J, Lehner R, Lenz G, Goldberg D (2005). Mental distress and quality of life in a deaf population. Soc Psychiatry Psychiatr Epidemiol.

[6] de Graaf R, Bijl RV (2002). Determinants of mental distress in adults with a severe auditory impairment: Differences between prelingual and postlingual deafness. Psychosom Med.

[7] Feldman DM, Gum A (2007). Multigenerational perceptions of mental health services of deaf adults in Florida. American Annals of the Deaf.

[8] Critchfield AB (2002). Meeting the Mental Health Needs of Persons Who Are Deaf.

[9] McKee MM, McKee K, Winters P, Sutter E, Pearson T (2014). Higher educational attainment but not higher income is protective for cardiovascular risk in Deaf American Sign Language (ASL) users. Disabil Health J.

[10] Alexander A, Ladd P, Powell S (2012). Deafness might damage your health. Lancet.

[11] Mitchell J, Howell C, Turnbull D, Murphy M (2005). Computer-assisted group therapy for the treatment of depression and anxiety in general practice. Prim Care Ment Health.

[12] Kushalnagar P, Holcomb J, Sadler G (2019). Genetic testing and eHealth usage among Deaf women. J Genet Couns.

[13] Pollard RQ, Betts WR, Carroll JK, Waxmonsky JA, Barnett S, deGruy FV, Pickler LL, Kellar-Guenther Y (2014). Integrating primary care and behavioral health with four special populations: Children with special needs, people with serious mental illness, refugees, and deaf people. Am Psychol.

[14] Landsberger S, Sajid A, Schmelkin L, Diaz D, Weiler C (2013). Assessment and treatment of deaf adults with psychiatric disorders: A review of the literature for practitioners. J Psychiatr Pract.

[15] Sudore RL, Landefeld CS, Pérez-Stable EJ, Bibbins-Domingo K, Williams BA, Schillinger D (2009). Unraveling the relationship between literacy, language proficiency, and patient-physician communication. Patient Educ Couns.

[16] Crowe TV (2017). Is telemental health services a viable alternative to traditional psychotherapy for deaf individuals?. Community Ment Health J.

[17] Wilson JAB, Schild S (2014). Provision of mental health care services to deaf individuals using telehealth. Prof Psychol Res Pr.

[18] Maiorana-Basas M, Pagliaro CM (2014). Technology use among adults who are deaf and hard of hearing: A national survey. J Deaf Stud Deaf Educ.

[19] Langarizadeh M, Tabatabaei M, Tavakol K, Naghipour M, Rostami A, Moghbeli F (2017). Telemental health care, an effective alternative to conventional mental care: A systematic review. Acta Inform Med.

[20] Behl DD, Kahn G (2015). Provider perspectives on telepractice for serving families of children who are deaf or hard of hearing. Int J Telerehab.

[21] O'Dea S (2022). Smartphone (consumer/enterprise) average price in the United States 2014-2025. Statista.

[22] Bitman N, John N (2019). Deaf and hard of hearing smartphone users: Intersectionality and the penetration of ableist communication norms. J Comput Mediated Commun.

[23] Romero RL, Kates F, Hart M, Ojeda A, Meirom I, Hardy S (2019). Quality of deaf and hard-of-hearing mobile apps: Evaluation using the Mobile App Rating Scale (MARS) with additional criteria from a content expert. JMIR Mhealth Uhealth.

[24] Balch G, Mertens D (1999). Focus group design and group dynamics: Lessons from deaf and hard of hearing participants. Am J Eval.

[25] Lipson SK, Kern A, Eisenberg D, Breland-Noble AM (2018). Mental health disparities among college students of color. J Adolesc Health.

[26] Venkatesh V, Thong JYL, Xu X (2012). Consumer acceptance and use of information technology: Extending the unified theory of acceptance and use of technology. MIS Q.

[27] Braun A, Clarke V (2006). Using thematic analysis in psychology. Qual Res Psychol.

[28] (2013). SAS® 9.4 — Today and Tomorrow. SAS Support.

[29] Padden C, Bauman D (2007). The decline of Deaf clubs in the US: A treatise on the problem of place. Sightings: Explorations in Deaf Studies.

[30] Gulliver A, Griffiths KM, Christensen H (2010). Perceived barriers and facilitators to mental health help-seeking in young people: A systematic review. BMC Psychiatry.

[31] Mata-Greve F, Johnson M, Pullmann M, Friedman E, Griffith Fillipo I, Comtois K, Arean P (2021). Mental health and the perceived usability of digital mental health tools among essential workers and people unemployed due to COVID-19: Cross-sectional survey study. JMIR Ment Health.

[32] Strecher V, McClure J, Alexander G, Chakraborty B, Nair V, Konkel J, Greene S, Couper M, Carlier C, Wiese C, Little R, Pomerleau C, Pomerleau O (2008). The role of engagement in a tailored web-based smoking cessation program: Randomized controlled trial. J Med Internet Res.

[33] Torous J, Nicholas J, Larsen ME, Firth J, Christensen H (2018). Clinical review of user engagement with mental health smartphone apps: Evidence, theory and improvements. Evid Based Ment Health.

[34] Clarkson P, Coleman R, Keates S, Lebbon C (2003). Inclusive Design: Design for the Whole Population.

[35] Borghouts J, Eikey E, Mark G, De Leon C, Schueller SM, Schneider M, Stadnick N, Zheng K, Mukamel D, Sorkin DH (2021). Barriers to and facilitators of user engagement with digital mental health interventions: Systematic review. J Med Internet Res.

[36] Steinberg A (1991). Issues in providing mental health services to hearing-impaired persons. Hosp Community Psychiatry.

[37] Steinberg AG, Barnett S, Meador HE, Wiggins EA, Zazove P (2006). Health care system accessibility. Experiences and perceptions of deaf people. J Gen Intern Med.

[38] Cabral L, Muhr K, Savageau J (2013). Perspectives of people who are deaf and hard of hearing on mental health, recovery, and peer support. Community Ment Health J.

[39] Steinberg AG, Sullivan VJ, Loew RC (1998). Cultural and linguistic barriers to mental health service access: The deaf consumer's perspective. Am J Psychiatry.

[40] Kern A, Hong V, Song J, Lipson SK, Eisenberg D (2018). Mental health apps in a college setting: Openness, usage, and attitudes. Mhealth.

[41] Diaz DR, Landsberger SA, Povlinski J, Sheward J, Sculley C (2013). Psychiatric disorder prevalence among deaf and hard-of-hearing outpatients. Compr Psychiatry.

[42] Black PA, Glickman NS (2006). Demographics, psychiatric diagnoses, and other characteristics of North American Deaf and hard-of-hearing inpatients. J Deaf Stud Deaf Educ.

