# Supplementary material for: Understanding the Potential of Mental Health Apps to Address Mental Health Needs of the Deaf and Hard of Hearing Community: Mixed Methods Study
Source: JMIR Hum Factors. 2022 Apr 11;9(2):e35641. doi: 10.2196/35641 (PMC9039808; doi:10.2196/35641)
Supplement: Multimedia Appendix 1 [file humanfactors_v9i2e35641_app1.docx]

Understanding the Potential of Mental Health Apps to Address Mental Health Needs of the Deaf and Hard of Hearing Community: Survey Items

Start of Block: General technology and smartphone use

This section is about your technology and smartphone use.

Q1 Which of the following do you use? (Select all that apply.)

- Desktop or laptop computer (1)
- Smartphone (2)
- Tablet (e.g., iPad) (5)
- Mobile phone or cell phone but not a smartphone (3)
- I don't use any of these. (4)

Display This Question:

If Which of the following do you use? (Select all that apply.) = Smartphone

Or Which of the following do you use? (Select all that apply.) = Mobile phone or cell phone but not a smartphone

Q2 *The California LifeLine Program is a state program that provides discounted home phones and cell phone services to eligible households.*
 
Do you participate in the California LifeLine program? (Select one answer.)

- Yes (1)
- No (2)
- I'm not sure. (3)
- I prefer not to answer. (4)

Q3 People may use the internet for streaming video/music, playing games, checking social media, using apps, browsing the web, etc. on a computer or on a phone or mobile device.
 
**BEFORE the pandemic (also known as COVID-19 or coronavirus) started:** On a typical day, how often did you use the internet? (Select one answer.)

- Almost constantly (1)
- Many times a day (2)
- A few times a day (3)
- Less than a few times a day (4)
- I prefer not to answer. (5)

| Page Break |  |
| --- | --- |

Q4
**SINCE the pandemic (also known as COVID-19 or coronavirus) started:** On a typical day, how often do you use the internet? (Select one answer.)

- Almost constantly (1)
- Many times a day (2)
- A few times a day (3)
- Less than a few times a day (4)
- I prefer not to answer. (5)

Q5 Where do you most often access internet? (Select one answer.)

- At home (1)
- At work (2)
- In public places (e.g., restaurant, library, etc.) (3)
- Other (4) ________________________________________________

Q6 Do you have consistent access to WiFi (e.g., wireless internet)? (Select one answer.)

- Yes (1)
- No (2)
- I’m not sure. (3)

| Page Break |  |
| --- | --- |

Display This Question:

If Which of the following do you use? (Select all that apply.) = Smartphone

Q7 Are you concerned about having enough space to download apps on your smartphone? (Select one answer.)

- Yes (1)
- No (2)
- I'm not sure. (3)

Q8 Do you have a mobile data plan (e.g., can you access internet on your phone without WiFi)? (Select one answer.)

- Yes (1)
- No (2)
- I'm not sure. (3)

| Page Break |  |
| --- | --- |

Display This Question:

If Do you have a mobile data plan (e.g., can you access internet on your phone without WiFi)? (Selec... = Yes

Q9 Do you have concerns about your mobile data plan when using your mobile device (for example, concerns that you will run out of data, an app will use up a lot of your data, etc.)? (Select one answer.)

- Yes (1)
- No (2)
- I'm not sure. (3)

Q10 Please rate the extent to which you agree or disagree, using a scale from **Strongly disagree (1) to Strongly agree (5).**

|  | Strongly disagree -1 (1) | Somewhat disagree - 2 (2) | Neither agree nor disagree - 3 (3) | Somewhat agree - 4 (4) | Strongly agree - 5 (5) |
| --- | --- | --- | --- | --- | --- |
| I am confident using technology to look up information. (1) |  |  |  |  |  |

End of Block: General technology and smartphone use

Start of Block: Healthcare utilization and resources

People use different resources and strategies to support their emotions and mental health. This part of the survey will look at the use of healthcare resources and other strategies to manage mental health.

Q11 What was the main reason you think the **Deaf and Hard of Hearing community** may not try to get help from an online tool, including mobile apps or texting services? (Select one answer.)

- Got better from other resources (1)
- Wanted to handle problem themselves (2)
- Don't own a smartphone or computer (3)
- Didn't know about these apps (4)
- Don't trust mobile apps (5)
- Concerns about privacy and security of data (6)
- Don't think it would be helpful or work (7)
- Cost (8)
- Don't have time (9)
- Received traditional / face-to-face services (10)
- Don't think they would need it (11)
- Don't have enough space to download new apps (12)
- Can't find an online tool that supports American Sign Language (ASL) (14)
- Other (Please specify): (13) ________________________________________________

| Page Break |  |
| --- | --- |

Q12 When thinking about using mental health resources, what things do you think the **Deaf and Hard of Hearing community** would you like to be able to do? (Select all that apply.)

- Identify or recognize symptoms (1)
- Track symptoms (2)
- Work through negative emotions and thoughts (3)
- Talk with other people to get / give support (4)
- Connect with a mental health professional (5)
- Read mental health experiences of other people (6)
- Stay organized and keep on track of tasks and responsibilities (7)
- Express themselves or have an outlet through art, photos, or writing (8)
- Distract themselves from negative thoughts or emotions (9)
- Get information about mental health symptoms and conditions (10)
- Get information about how to access local mental health resources (11)
- Access educational materials on how to cope with stress (12)
- Get information about how to handle grief or loss (13)
- Get information about how to deal with trauma (14)
- Get information about how to handle relationship issues (15)
- Other (Please specify): (16) ________________________________________________

| Page Break |  |
| --- | --- |

Q13 What barriers, if any, do you think the **Deaf and Hard of Hearing community**faces to accessing mental health-related resources? (Select all that apply.)

- They prefer to deal with issues on their own (2)
- They don’t have time (4)
- The waiting time to access resources is too long (5)
- There are financial reasons (too expensive, no insurance) (3)
- Hard to find mental health care providers that know American Sign Language (ASL) (23)
- Lack of access to an interpreter (19)
- Even if an interpreter can be provided, they don't feel comfortable with having an interpreter present (24)
- They are concerned about privacy (6)
- They worry about what others will think of them (8)
- People providing services aren’t sensitive enough to cultural differences (10)
- People providing services aren’t sensitive enough to gender or sexual identity differences (11)
- They have a hard time communicating in English (12)
- They question the quality of their options (13)
- They question whether the resources are helpful (14)
- They have had a bad experience with these resources in the past (15)
- The problem will get better by itself (16)
- They question how serious their needs are (17)
- They don’t think anyone can understand their problems (18)
- They get a lot of support from other sources (20)
- There have been no barriers or challenges that I can think of (21)
- Other (Please specify): (22) ________________________________________________
- They have not had any need for any mental health resources (7)

| Page Break |  |
| --- | --- |

Q14 The next set of statements are going to ask you about resources and strategies**you** use or would like to use to manage your mental health. For each statement, select if you currently use it or if you would like to use it.

|  | I currently use this resource. (4) | I would like to use this resource. (5) |
| --- | --- | --- |
| Informal support, such as talking with or spending time with family or friends (1) |  |  |
| Peer support, such as talking with people who are similar to me (7) |  |  |
| Professional services (examples: counseling with psychologist, clinical social worker, psychiatrist) (2) |  |  |
| Social media (examples: Facebook, Twitter, Reddit) (3) |  |  |
| Online forums or communities (examples: Mental Health Forum, BeyondBlue) (4) |  |  |
| Websites (examples: Moodgym, Psychology Today) (5) |  |  |
| Mobile apps (examples: 7 Cups, Headspace, Moodpath) (6) |  |  |
| Riverside TakeMyHand online chat (22) |  |  |
| Exercise programs or physical activities (8) |  |  |
| Activities like writing, painting, playing or making music (13) |  |  |
| Other (Please Specify): (21) |  |  |

| Page Break |  |
| --- | --- |

Display This Question:

If The next set of statements are going to ask you about resources and strategies you use or would l... != I currently use this resource.

Q14a In the previous question, you didn't select any resources that you **currently use**. Do you **currently use** any resources to manage your mental health? (Select one answer.)

- Yes (1)
- No (3)

| Page Break |  |
| --- | --- |

Display This Question:

If In the previous question, you didn't select any resources that you currently use. Do you currentl... = Yes

Q14_b The next set of statements are going to ask you about resources and strategies**you currently** use to manage your mental health. For each statement, select if you currently use it.

|  | I currently use this resource. (4) |
| --- | --- |
| Informal support, such as talking with or spending time with family or friends (1) |  |
| Peer support, such as talking with people who are similar to me (7) |  |
| Professional services (examples: counseling with psychologist, clinical social worker, psychiatrist) (2) |  |
| Social media (examples: Facebook, Twitter, Reddit) (3) |  |
| Online forums or communities (examples: Mental Health Forum, BeyondBlue) (4) |  |
| Websites (examples: Moodgym, Psychology Today) (5) |  |
| Mobile apps (examples: 7 Cups, Headspace, Moodpath) (6) |  |
| Riverside TakeMyHand online chat (22) |  |
| Exercise programs or physical activities (8) |  |
| Activities like writing, painting, playing or making music (13) |  |
| Other (Please Specify): (21) |  |

| Page Break |  |
| --- | --- |

Display This Question:

If The next set of statements are going to ask you about resources and strategies you use or would l... != I would like to use this resource.

Q14c In the previous question, you didn't select any resources **you would like to use**. Are you interested in any other resources to manage your mental health? (Select one answer)

- Yes (1)
- No (2)

| Page Break |  |
| --- | --- |

Display This Question:

If In the previous question, you didn't select any resources you would like to use. Are you interest... = Yes

Q14_d The next set of statements are going to ask you about resources and strategies**you would like to use** to manage your mental health. For each statement, select if you would like to use it.

|  | I would like to use this resource. (4) |
| --- | --- |
| Informal support, such as talking with or spending time with family or friends (1) |  |
| Peer support, such as talking with people who are similar to me (7) |  |
| Professional services (examples: counseling with psychologist, clinical social worker, psychiatrist) (2) |  |
| Social media (examples: Facebook, Twitter, Reddit) (3) |  |
| Online forums or communities (examples: Mental Health Forum, BeyondBlue) (4) |  |
| Websites (examples: Moodgym, Psychology Today) (5) |  |
| Mobile apps (examples: 7 Cups, Headspace, Moodpath) (6) |  |
| Riverside TakeMyHand online chat (22) |  |
| Exercise programs or physical activities (8) |  |
| Activities like writing, painting, playing or making music (13) |  |
| Other (Please Specify): (21) |  |

End of Block: Healthcare utilization and resources

Start of Block: Mental Health Perceptions

Q15 The following questions are going to ask you about your personal thoughts and opinions about **those with a mental illness.** Throughout this section, the term "mental illness" will be used. However, there are other terms that could be used, such as mental disorder, psychological disorder, or mental health condition. 

 Please rate the extent to which you agree, on a scale from **Strongly Disagree (1) to Strongly Agree (4)** for the following statements.

|  | Strongly disagree-1 (1) | Disagree-2 (2) | Agree-3 (3) | Strongly agree-4 (4) |
| --- | --- | --- | --- | --- |
| Most people believe that having a mental illness is a sign of personal weakness. (2) |  |  |  |  |
| Most people believe that people with a mental illness are dangerous. (4) |  |  |  |  |
| If they had a mental illness, most people would not tell anyone. (7) |  |  |  |  |

End of Block: Mental Health Perceptions

Start of Block: Mental health apps

This section is about technology specifically for mental health.

Q16 Have you ever used a mental health app? (Select one answer.) *By mental health app, we mean an application on your mobile phone or tablet device that helps you manage your mental, emotional, or psychological health or get access to resources to support your mental, emotional, or psychological health.*

- Yes, I currently use a mental health app. (4)
- Yes, I have used a mental health app but no longer use one. (5)
- No, I have never used a mental health app, but I am interested in using one. (6)
- No, I have never used a mental health app, but I am not interested in using one. (9)

Display This Question:

If Have you ever used a mental health app? (Select one answer.)   By mental health app, we mean an a... = Yes, I currently use a mental health app.

Or Have you ever used a mental health app? (Select one answer.)   By mental health app, we mean an a... = Yes, I have used a mental health app but no longer use one.

Q17 The next set of statements are going to ask you about your views on using mental health apps. For each statement, please rate the extent to which you agree or disagree, using a scale from **Strongly disagree (1) to Strongly agree (5).** *By mental health app, we mean an application on your mobile phone or tablet device that helps you manage your mental, emotional, or psychological health or get access to resources to support your mental, emotional, or psychological health.*

|  | Strongly disagree- 1 (1) | Somewhat disagree- 2 (2) | Neither agree nor disagree- 3 (3) | Somewhat agree- 4 (4) | Strongly agree- 5 (5) |
| --- | --- | --- | --- | --- | --- |
| I find mental health apps useful in my daily life. (1) |  |  |  |  |  |

| Page Break |  |
| --- | --- |

Q18 The next set of statements are going to ask you about your views on mental health apps. For each statement, please rate the extent to which you agree or disagree, using a scale from **Strongly disagree (1) to Strongly agree (5)**.
 
*By mental health app, we mean an application on your mobile phone or tablet device that helps you manage your mental, emotional, or psychological health or get access to resources to support your mental, emotional, or psychological health.*

|  | Strongly disagree- 1 (1) | Somewhat disagree- 2 (2) | Neither agree nor disagree- 3 (3) | Somewhat agree- 4 (4) | Strongly agree- 5 (5) |
| --- | --- | --- | --- | --- | --- |
| I have the resources necessary to use mental health apps. (5) |  |  |  |  |  |
| Mental health apps are compatible with other technologies I use. (6) |  |  |  |  |  |
| I can get help from others when I have difficulties using mental health apps. (7) |  |  |  |  |  |

| Page Break |  |
| --- | --- |

Display This Question:

If Have you ever used a mental health app? (Select one answer.)   By mental health app, we mean an a... = Yes, I currently use a mental health app.

Or Have you ever used a mental health app? (Select one answer.)   By mental health app, we mean an a... = Yes, I have used a mental health app but no longer use one.

Q19_used The next set of statements are going to ask you about your views on how your personal information might be used by a mental health app. For each statement, please rate the extent to which you agree or disagree on a scale from **Strongly disagree (1) to Strongly agree (5)**.

*By mental health app, we mean an application on your mobile phone or tablet device that helps you manage your mental, emotional, or psychological health or get access to resources to support your mental, emotional, or psychological health.*

|  | Strongly disagree-1 (1) | Somewhat disagree-2 (2) | Neither agree nor disagree-3 (3) | Somewhat agree-4 (4) | Strongly agree-5 (5) |
| --- | --- | --- | --- | --- | --- |
| I feel that as a result of my using mental health apps, others know about me more than I am comfortable with. (1) |  |  |  |  |  |

Display This Question:

If Have you ever used a mental health app? (Select one answer.)   By mental health app, we mean an a... = No, I have never used a mental health app, but I am <u>interested</u> in using one.

Or Have you ever used a mental health app? (Select one answer.)   By mental health app, we mean an a... = No, I have never used a mental health app, but I am<u> not interested</u> in using one.

Q19_never used The next set of statements are going to ask you about your views on how your personal information might be used by a mental health app. For each statement, please rate the extent to which you agree or disagree on a scale from **Strongly disagree (1) to Strongly agree (5)**.

By mental health app, we mean an application on your mobile phone or tablet device that helps you manage your mental, emotional, or psychological health or get access to resources to support your mental, emotional, or psychological health. 

|  | Strongly disagree-1 (1) | Somewhat disagree-2 (2) | Neither agree nor disagree-3 (3) | Somewhat agree-4 (4) | Strongly agree-5 (5) |
| --- | --- | --- | --- | --- | --- |
| I feel that if I were to use mental health apps, others know about me more than I am comfortable with. (1) |  |  |  |  |  |

| Page Break |  |
| --- | --- |

Q20 When thinking about using mental health apps, what other aspects are important to you? (Select all that apply.)

By mental health app, we mean an application on your mobile phone or tablet device that helps you manage your mental, emotional, or psychological health or get access to resources to support your mental, emotional, or psychological health. 

- The app supports American Sign Language (ASL) (13)
- The app includes many visual elements, such as pictures and videos, rather than text (14)
- Availability in languages other than English (1)
- The app is free (2)
- The app is sensitive to my culture (3)
- People I interact with on the app share the same cultural background as I do (4)
- People I interact with on the app share similar mental health experience as I do (5)
- My personal information will be kept private (6)
- The app will not have a negative effect on my device (examples: using the app will not drain my phone battery, using the app will not take up too much memory) (7)
- Parts of the app can be used offline (8)
- The app can be easily used by people with visual impairments (9)
- Other (Please specify) (11) ________________________________________________

Q21 The next set of statements are going to ask you about how important each of the following are to you in an app for mental health, on a scale from **Not at all important (1) to Extermely important (5).**

|  | Not at all important -1 (18) | Slightly important -2 (19) | Moderately important -3 (20) | Very important -4 (21) | Extremely important -5 (22) |
| --- | --- | --- | --- | --- | --- |
| Referral to clinical mental health services outside of the app (1) |  |  |  |  |  |
| Telehealth: Direct connection to clinical mental health services within the app (examples: psychologist, clinical social worker, therapist, psychiatrist) (2) |  |  |  |  |  |
| Peer support and chat (3) |  |  |  |  |  |
| Education or information on how to spot the signs and symptoms of mental illness, causes, and treatments (4) |  |  |  |  |  |
| Self-tracking mental health symptoms, such as mood and emotions (5) |  |  |  |  |  |
| Crisis or emergency support (6) |  |  |  |  |  |
| Mental illness screening (7) |  |  |  |  |  |
| Suicide prevention (8) |  |  |  |  |  |
| Artificial intelligence or chatbot *(Some apps can copy how a human talks and act like a real-life conversation, though it’s actually a computer. In mental health apps, chatbots usually help people talk through how they are feeling by asking questions.)* (9) |  |  |  |  |  |

| Page Break |  |
| --- | --- |

Q22 Please rank the **top three** things that are most important to you in a mental health app. 


To rank the items, drag one item on the left into each of the three boxes on the right.

| Top or First Choice | Second Choice | Third Choice |
| --- | --- | --- |
| ______ Referral to clinical mental health services outside of app (1) | ______ Referral to clinical mental health services outside of app (1) | ______ Referral to clinical mental health services outside of app (1) |
| ______ Telehealth: Direct connection to clinical mental health services within the app (examples: psychologist, clinical social worker, therapist, psychiatrist) (2) | ______ Telehealth: Direct connection to clinical mental health services within the app (examples: psychologist, clinical social worker, therapist, psychiatrist) (2) | ______ Telehealth: Direct connection to clinical mental health services within the app (examples: psychologist, clinical social worker, therapist, psychiatrist) (2) |
| ______ Peer support and chat (3) | ______ Peer support and chat (3) | ______ Peer support and chat (3) |
| ______ Education or information on how to spot the signs and symptoms of mental illness, causes, and treatments (4) | ______ Education or information on how to spot the signs and symptoms of mental illness, causes, and treatments (4) | ______ Education or information on how to spot the signs and symptoms of mental illness, causes, and treatments (4) |
| ______ Self-tracking mental health symptoms, such as mood and emotions (5) | ______ Self-tracking mental health symptoms, such as mood and emotions (5) | ______ Self-tracking mental health symptoms, such as mood and emotions (5) |
| ______ Crisis or emergency support (6) | ______ Crisis or emergency support (6) | ______ Crisis or emergency support (6) |
| ______ Mental illness screening (7) | ______ Mental illness screening (7) | ______ Mental illness screening (7) |
| ______ Suicide prevention (8) | ______ Suicide prevention (8) | ______ Suicide prevention (8) |
| ______ Artificial intelligence or chatbot (Some apps can copy how a human talks and act like a real-life conversation, though it's actually a computer. In mental health apps, chatbots usually help people talk through how they are feeling by asking questions.) (9) | ______ Artificial intelligence or chatbot (Some apps can copy how a human talks and act like a real-life conversation, though it's actually a computer. In mental health apps, chatbots usually help people talk through how they are feeling by asking questions.) (9) | ______ Artificial intelligence or chatbot (Some apps can copy how a human talks and act like a real-life conversation, though it's actually a computer. In mental health apps, chatbots usually help people talk through how they are feeling by asking questions.) (9) |

| Page Break |  |
| --- | --- |

Q23 The next set of statements are going to ask you how important each of the following content areas are to you in an app for mental health, on a scale from **Not at all important (1) to Extremely important (5).**

|  | Not at all important -1 (59) | Slightly important -2 (60) | Moderately important -3 (61) | Very important -4 (62) | Extremely important -5 (63) |
| --- | --- | --- | --- | --- | --- |
| Depression (9) |  |  |  |  |  |
| Anxiety (10) |  |  |  |  |  |
| Schizophrenia (11) |  |  |  |  |  |
| Obsessive compulsive disorder (OCD) (12) |  |  |  |  |  |
| Bipolar disorder (13) |  |  |  |  |  |
| Substance and/or alcohol use (14) |  |  |  |  |  |
| Eating disorder (15) |  |  |  |  |  |
| Loneliness (16) |  |  |  |  |  |
| Dealing with a life event (examples: moving, death, illness) (17) |  |  |  |  |  |
| Mindfulness and stress reduction (18) |  |  |  |  |  |
| Other (please specify): (8) |  |  |  |  |  |

End of Block: Mental health apps

Start of Block: Personal experiences

This part of the survey asks about your own experiences. You are the experts, and we are here to learn. The purpose of these questions is to learn from you and your preferences so that we can better serve the Deaf and Hard of Hearing community.

Q24 There are many different ways people identify. For example, some people may identify with Deaf culture or refer to themselves as Hard of Hearing. We know the experiences of each person are different. How do you identify?

________________________________________________________________

Q25 Which statement best describes your hearing?

- Full hearing (1)
- Some hearing (2)
- No hearing (3)
- Other (please specify): (4) ________________________________________________

Q26 How old were you when you began to have any hearing loss?

________________________________________________________________

Q27 What is your preferred communication method? (Select one.)

- American Sign Language (ASL) (1)
- Another Sign Language (please specify): (4) ________________________________________________
- Speaking (5)
- Reading and writing (6)
- Other (please specify): (7) ________________________________________________

| Page Break |  |
| --- | --- |

Q28 What else would you like us to know? 
This includes but is not limited to sharing more information about Deaf culture; mental health or wellness; coping strategies and resources; or use of and interest in technology for mental health.  *If you prefer not to answer, then you can leave this blank.*

________________________________________________________________

End of Block: Personal experiences

Start of Block: Demographics

This last part of the survey asks you about your background and demographic information. We use this information to understand who this data represents to make sure we best serve you and the community.

Q29 How old are you?

________________________________________________________________

Q30 What is your gender? (Select one answer.)

- Man (1)
- Woman (2)
- Transgender man (3)
- Transgender woman (4)
- Genderqueer / Gender non-conforming / Non-binary (5)
- Questioning or unsure of gender identity (6)
- Another gender identity (7) ________________________________________________
- I prefer not to answer. (8)

Q31 What is your race? (Select one answer.)

- American Indian or Alaska Native (1)
- Asian (2)
- Black or African American (3)
- Native Hawaiian or other Pacific Islander (4)
- White (5)
- Other: (6) ________________________________________________
- More than one race (7)
- I prefer not to answer. (8)

Q32 What is your ethnicity? (Select one answer. If you are multi-ethnic, please check "more than one ethnicity.")

- Caribbean (1)
- Central American (2)
- Mexican/Mexican-American/Chicano (3)
- Puerto Rican (4)
- South American (5)
- Other (6) ________________________________________________
- African (7)
- Asian Indian/South Asian (8)
- Cambodian (9)
- Chinese (10)
- Eastern European (11)
- European (12)
- Filipino (13)
- Japanese (14)
- Korean (15)
- Middle Eastern (16)
- Vietnamese (17)
- Other (18) ________________________________________________
- More than one ethnicity (19)
- I prefer not to answer. (20)

Q33 What is your sexual orientation? (Select one answer.)

- Gay or Lesbian (1)
- Heterosexual or Straight (2)
- Bisexual (3)
- Questioning or unsure of sexual orientation (4)
- Queer (5)
- Another sexual orientation: (6) ________________________________________________
- I prefer not to answer. (7)

Q34 What language do you most often speak at home? (Select one answer.)

- American Sign Language (ASL) (19)
- Another Sign Language (please specify) (20) ________________________________________________
- Arabic (1)
- Armenian (2)
- Cambodian (3)
- Cantonese (4)
- English (5)
- Farsi (6)
- Hmong (7)
- Korean (8)
- Mandarin (9)
- Other Chinese (10)
- Russian (11)
- Spanish (12)
- Tagalog (13)
- Vietnamese (14)
- American Sign Language (15)
- Other: (16) ________________________________________________
- I prefer not to answer. (17)

End of Block: Demographics

Start of Block: End of Survey
